# Supplementary material for: Exercise interventions in migraine patients: a YouTube content analysis study based on grades of recommendation
Source: PeerJ. 2022 Sep 30;10:e14150. doi: 10.7717/peerj.14150 (PMC9528906; doi:10.7717/peerj.14150)
Supplement: Supplemental Information 4 [file peerj-10-14150-s004.docx]

**CODEBOOK**

**Descriptive data of YT videos**

1. GQS: Global Quality Scale
2. DISCERN_Total: total score of DISCERN Scale
3. Video_Power_Index: like count/(like count + dislike count) × 100
4. Ratio_Visits_per_Day: view count/days online
5. Author dichotomized: grouped authors into two categories (professional authors and non-professional authors)

**Percentage of videos regarding GR**

1. A (IFT): grade A In Favor of Treatment
2. B (IFT): grade B In Favor of Treatment
3. C (IFT): grade C In Favor of Treatment
4. D (IFT): grade D In Favor of Treatment
5. C (AT): grade C Against Treatment
